# Two cases of breast angiosarcoma after breast conserving surgery

**DOI:** 10.1186/s40792-020-00841-w

**Published:** 2020-04-26

**Authors:** Eriko Shiraki, Yookija Kang, Takahiro Shibayama, Shigeru Tsuyuki

**Affiliations:** 1 Department of Breast Surgery, Osaka Red Cross Hospital, 5-30 Fudegasaki-cho, Tennoji-ku, Osaka, 543-8555 Japan; 2 Department of Diagnostic Pathology, Osaka Red Cross Hospital, 5-30 Fudegasaki-cho, Tennoji-ku, Osaka, 543-8555 Japan

**Keywords:** Primary angiosarcoma, Radiation-induced angiosarcoma, Breast cancer, Breast conserving surgery, Surgical margin

## Abstract

**Background:**

Breast angiosarcoma (AS) is a rare malignant breast tumor arising from endothelial cells lining the blood vessels. The prognosis of AS is reportedly poor. However, the effectiveness of chemotherapy and radiation is still controversial. Surgery is the only curable treatment, and removal of AS with adequate surgical margin is important.

**Case presentation:**

We report two cases of primary and radiation-induced breast angiosarcoma (AS) after performing breast conserving surgery (BCS) for breast cancer. In case 1, a 72-year-old woman underwent right BCS with adjuvant radiation therapy (RT) for breast cancer 5 years prior. She was diagnosed with AS of the right breast and underwent mastectomy with a wide skin resection of the breast. As the tumor cells were positive for c-myc, this tumor was diagnosed as a radiation-induced AS. In case 2, an 80-year-old woman underwent BCS without adjuvant RT. She was diagnosed with AS 3 years after BCS and underwent mastectomy with a wide skin resection of the breast. The tumor was diagnosed to be a primary AS because there were no episodes of RT or lymphedema. Both cases developed local recurrence within 1 year of surgery.

**Conclusion:**

Our cases suggest that surgical margin is associated with the risk of local recurrence, and the difficulty of deciding a safe surgical margin should be set during preoperative diagnosis.

## Background

Breast angiosarcoma (AS) is a rare malignancy arising from the endothelial cells lining the blood vessels and accounts for 0.05% of all malignant breast tumors. It can be divided into primary and secondary AS. Secondary AS occurs after adjuvant radiation therapy (RT) for breast cancer or chronic lymphedema. The prognosis of AS is reportedly poor. The efficacy of chemotherapy for breast AS was reported in several trials such as the AngioTax study, which demonstrated that a weekly paclitaxel regimen was effective for metastatic or unresectable angiosarcoma. However, the recommended treatment for AS is surgical resection.

We report two cases of breast AS considered as primary and secondary tumors, respectively, after breast-conserving surgery (BCS) that had an early local recurrence 1 year after the surgery. We experienced the difficulty of radical surgery to reduce the risk of local recurrence in these cases.

### Case report 1

**Fig. 1:**
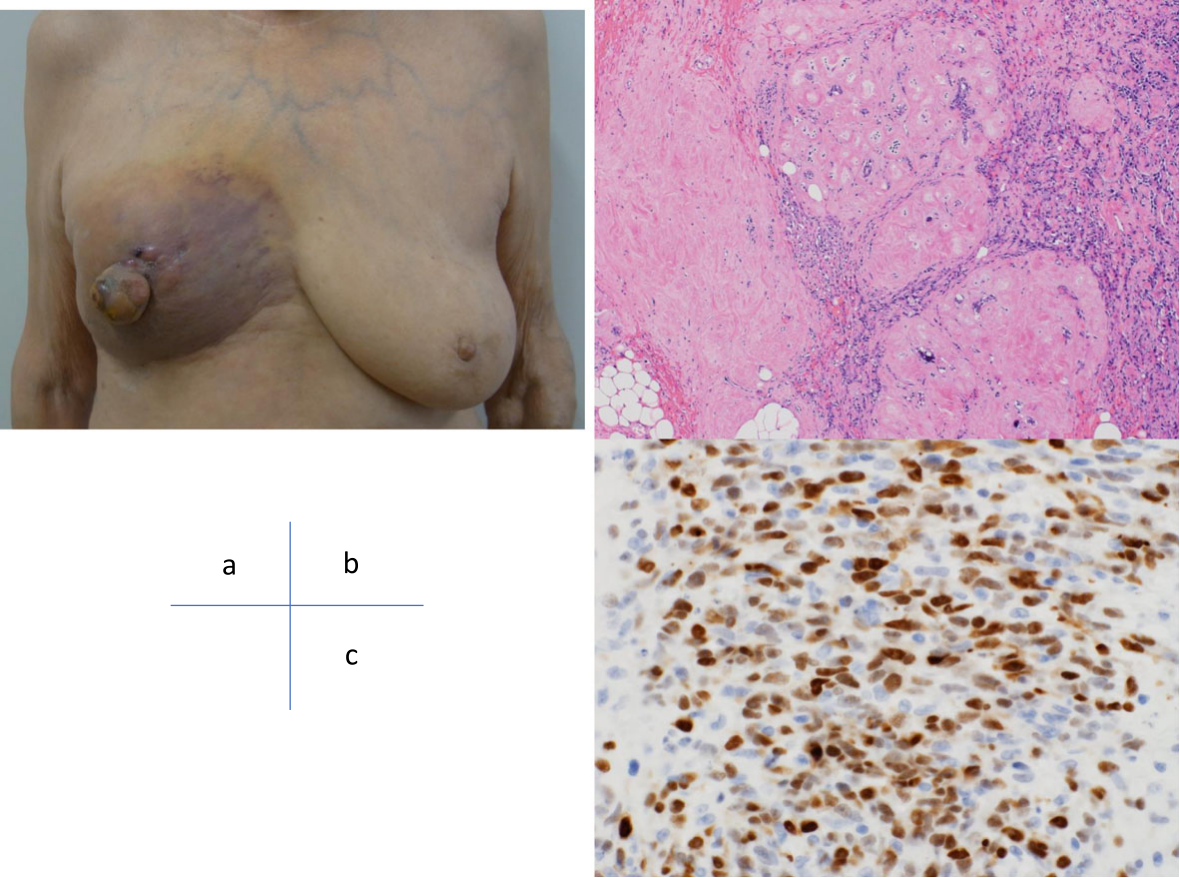
**a** Nipple swelling and reddening in of the right breast 5 years after right breast conserving therapy and adjuvant radiotherapy. Microscopic appearance of the angiosarcoma. **b** Neoplastic cells were spread around the irradiated atrophic breast tissue (× 40 magnification) and **c** Most of the spindle-shaped tumor cells expressed c-myc (× 400 magnification)

**Fig. 2:**
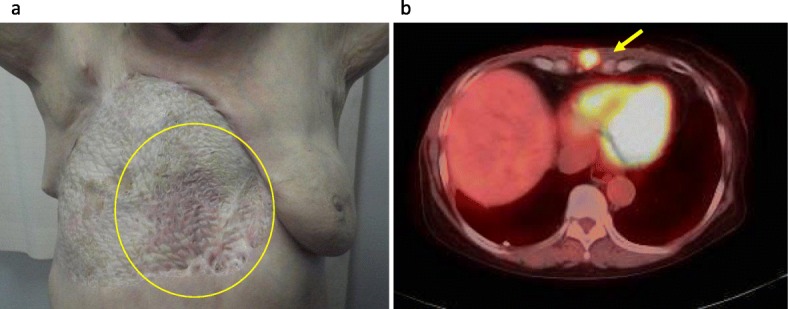
One year after breast surgery, the following two recurrences appeared: **a** Local recurrence of the skin graft (circled) and **b** Intramuscular metastatic tumor near the xiphoid process (indicated by an arrow)

A 72-year-old woman who underwent right BCS for breast cancer (invasive ductal carcinoma (IDC), ER+, PR+, HER2-, T1bN0M0) 5 years previously complained of nipple swelling and reddening of the right breast during a follow-up visit (Fig. 1a). Adjuvant RT (50 Gy) for residual breast tissue and adjuvant endocrine therapy were performed for 5 years. The ultrasound examination revealed a 24 × 15 × 13-mm low echoic area in the right breast. AS was diagnosed by punch skin biopsy. No metastasis to other organs was observed by a computed tomography (CT) scan. Right mastectomy with extensive skin resection with a margin of 3 cm from the discolored lesion and skin graft surgery was performed. The intraoperative rapid diagnosis of the resection margin was performed at several points. All points of the margin revealed no tumor cells. High grade AS was diagnosed from the final pathological findings. The size of the intramammary tumor was 18 × 15 cm, and the excisional margin of the skin was negative for tumor cells. There were spindle-shaped tumor cells in which CD34, factor VIII, and c-myc were locally positive, suggesting radiation-induced AS (Fig. 1b, c). One year after the surgery, the patient developed skin redness on the skin graft and an intramuscular tumor near the xiphoid process (Fig. 2). The skin biopsy confirmed the recurrence of AS. The patient received treatment for recurrence at another hospital. She received weekly paclitaxel (45 mg/m^2^) as first-line treatment. Because the patient had chronic renal failure requiring blood dialysis, a 40% dose reduction of paclitaxel was needed. A complete clinical response was achieved, which persisted for 11 months. Owing to new skin metastases, she received trabectedin as second-line treatment. Unfortunately, she died 32 months after surgery.

### Case report 2

**Fig. 3:**
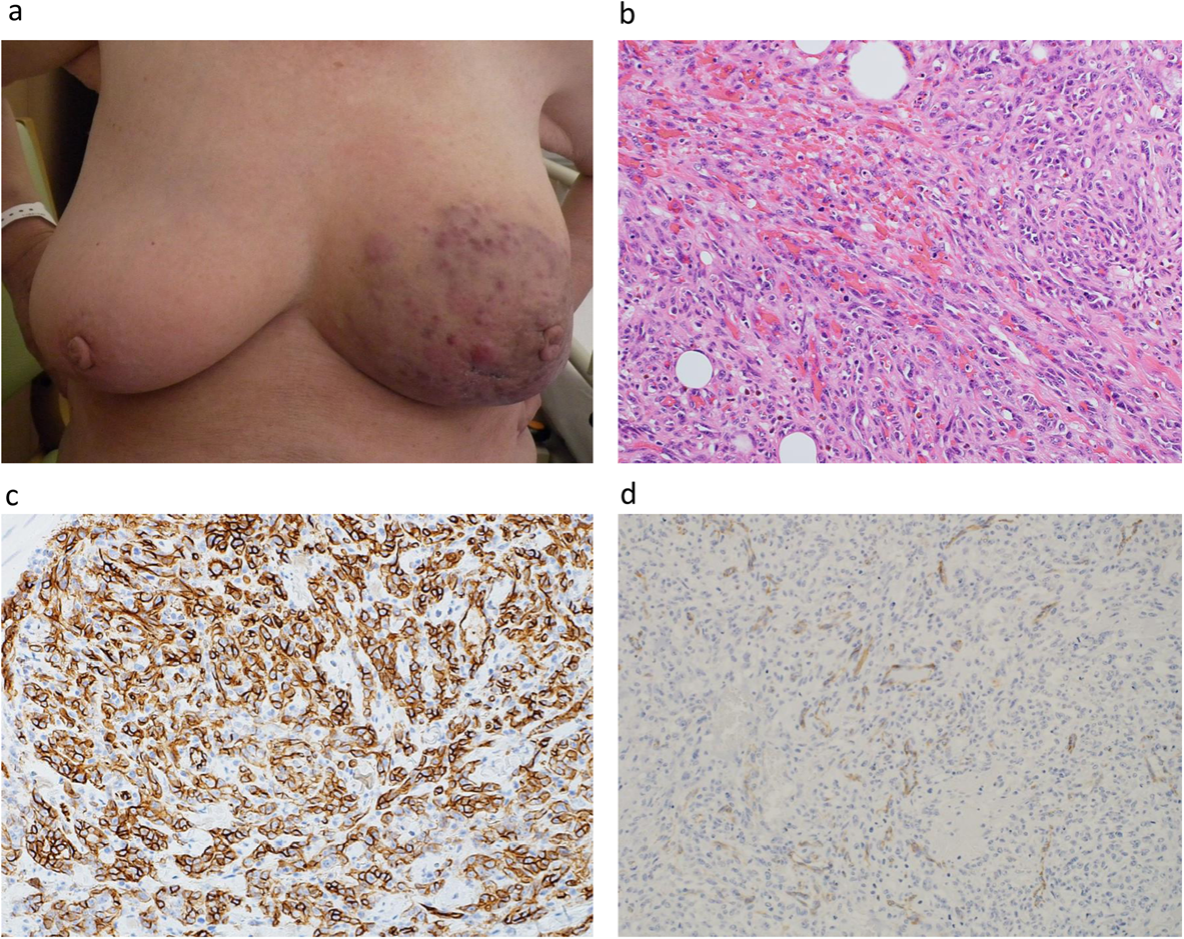
**a T**he skin nodules and color changes on the inferior side of the left breast 3 years after right breast conserving therapy. **b** Microscopic examination showed a solid hemorrhagic tumor composed of spindle-shaped cells with abundant mitosis (× 100 magnification). **c**, **d** Tumor cells were CD34-positive (**c** × 100 magnification) and focally positive for CD31 (**d** × 200 magnification)

**Fig. 4:**
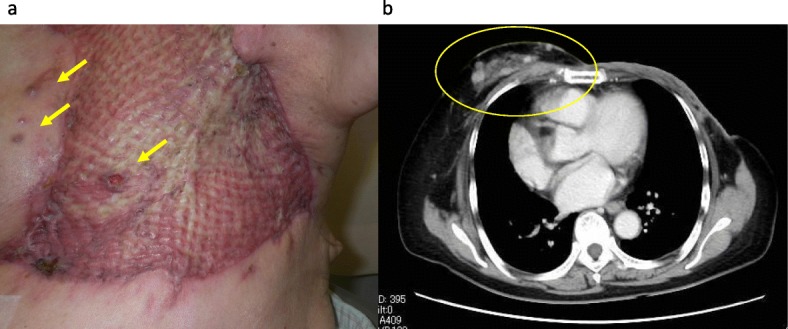
The following two recurrences appeared 40 days after surgery: **a** Local recurrence on the skin graft and skin metastases of the contralateral breast (indicated by arrows) and **b** Enhanced nodules in the contralateral breast as detected by MRI (circled)

An 80-year-old woman who underwent left BCS for breast cancer (IDC, ER+, PR+, HER2-, T2N0M0) 3 years previously complained of skin nodules and color changes on the inferior side of her left breast during a follow-up visit (Fig. 3a). She was receiving endocrine therapy scheduled for 5 years. Adjuvant RT was omitted because of her old age. The red skin nodules on the left breast were diagnosed as AS by punch biopsy. Magnetic resonance imaging (MRI) revealed solid and hyper-vascularized components with significant enhancement throughout the left breast. No metastasis was observed in other organs. Left mastectomy with extensive skin resection with a margin of 3 cm from the discolored lesion and skin graft surgery was performed. An additional skin resection of 2 cm was performed because several points of the resected stump were diagnosed as positive for tumor cells by intraoperative rapid diagnosis. Pathological findings revealed high grade AS. CD34 and CD31+ spindle tumor cells were detected, and the high-grade tumor cells had a high Ki67 index score of 80% (Fig. 3b–d). The high grade tumor cells were locally positive for c-myc although the patient never received RT. Unfortunately, the low grade tumor cells were widely detected in the lateral and deep margins, suggesting that the AS had spread beyond the prediction of the preoperative diagnosis from the MRI. Curative resection was considered impossible, and we decided to follow up without additional resection. Forty days after the surgery, skin nodules and redness appeared on the skin of the contralateral breast and on the skin graft of the chest wall (Fig. 4a). The skin biopsy diagnosed the recurrence of AS. CT scan revealed recurrent AS extending over the contralateral breast and chest wall (Fig. 4b). The patient received treatment for recurrence at another hospital. The first-line regimen comprised weekly paclitaxel 70 mg/m^2^. A dose reduction of paclitaxel was needed because she was 80 years old. She now has maintained clinical partial response 17 months after surgery.

## Discussion

We reported two cases of breast AS: a case of radiation-related AS and another of possible primary breast AS. Unfortunately, local recurrence developed in both patients within 1 year although both patients received mastectomy with wide skin resection.

AS usually appears as a soft breast tumor which grows rapidly with changes in skin color and the occurrence of purple skin nodules on the breast. It often involves diffuse and multifocal tumor nodules in the whole breast. Breast AS can be divided into primary and secondary tumors. Factors that predispose individuals to secondary AS include RT, Stewart-Treves syndrome, and chronic lymphedema [1–3]. Radiation-induced AS is being reported with increasing frequency. Though primary AS of the breast only makes up 0.04% of all breast malignancies, the frequency of AS increases to 0.16% after radiotherapy [4].

Arlen et al. defined radiation-induced AS as tumors that develop in patients with a history of RT at least 3 years before the occurrence of the tumor, occur on the irradiated area, and are pathologically different from past breast cancers [5]. Recently, Lae et al. reported that radiation-induced AS showed higher expression of c-myc than primary AS [6]. Case 1 matched all criteria of secondary AS. On the other hand, case 2 did not have a history of either RT or lymphedema, so she was diagnosed as having primary AS. Nevertheless, she was older, and her tumor cells were focally positive for c-myc, suggesting the possibility that it might be secondary AS.

Some clinicopathological features may influence the survival outcome of breast sarcoma including AS, such as tumor grade, tumor size, tumor spread, and margin status. Yin et al. reported that an older age, higher grade of tumor, and regional spread were prognostic factors of poor overall survival, and that the median overall survival was 93 and 32 months, and the 5-year survival rates were 44.5% and 22.5% for primary and secondary breast AS [7, 8].

Surgery represents the only potentially curative modality for breast AS. Adjuvant chemotherapy and radiation are still controversial. Total mastectomy with adequate surgical margin is generally performed as standard treatment. In a study of 100 cases of secondary breast AS treated with BCS, it was reported that over 73% of patients developed local recurrence, the majority of which occurred within 1 year [9]. AS often develops over a much wider field of the breast or chest wall than anticipated. Several studies reported that an adequate surgical margin was associated with a reduction of local recurrence and improved overall survival. Li et al. reported that radical resection of all or nearly all previously irradiated skin plus mastectomy reduced the local recurrence rate and improved 5-year disease-specific survival, compared with mastectomy/wide excision with partial skin resection [10]. The following two papers reported the relationship between clinical lateral margin and local recurrence. Jallali et al. studied 12 patients with radiation-induced AS who had surgery as primary treatment [11]. Their tumors were excised with a minimum macroscopic margin of 3 cm. It was demonstrated that no patient survived beyond 15 months when complete excision (complete histological margin taken to be > 10 mm) of the primary tumor was not achieved, while patients in whom a complete excision was achieved had a median survival of 42 months. This suggested that local control may be important in determining long-term outcome. Lindford et al. studied 8 patients who underwent excision with marked macroscopic lateral margin widths ranging from 3 to 5 cm [12]. Of the 8 patients, 2 developed local recurrence on the skin within the irradiated fields close to the earlier excision edge within 3 months of surgery and finally died from disseminated AS. The authors recommended performing the resection with macroscopic lateral margins greater than 3 cm, preferably of 4–5 cm and with deep margins including at least the pectoralis fascia and including the extent of the irradiated field to the surgical margin widths.

Our two cases developed local recurrence within 1 year after surgery. In case 2 of our study, we performed mastectomy with extensive skin resection, including the pectoralis fascia, but the margin tested positive for tumor cells because of the deep stump of the tumor, resulting in early local recurrence at 40 days after surgery. Although we retrospectively reviewed the images from the ultrasound and MRI after the surgery, it was difficult to make a diagnosis for the surgical margin. We were aware of the difficulty of preoperative diagnosis for the safety surgical margin.

On the other hand, case 1 developed recurrence on the skin graft and intramuscular recurrence at 1 year after surgery, although no tumor cells were found during resection of the stump. Although the local recurrence may be associated with the positivity of the resected stump, other factors may be associated with the recurrence pattern of case 1. Although hematogenous metastasis may have occurred at both recurrence sites, direct invasion from the main tumor leading to the recurrence on the skin graft is possible because the main tumor was large, and the tumor edge was close to the pectoral muscle. Li et al. reported better prognosis after radical surgery than after conservative surgery in radiation-induced AS if the pathological surgical margins were negative. Thus, case 1 may undergo a more radical surgery such as that including partial resection of the pectoral muscle to obtain negative surgical margins.

There is no standard systemic therapy for unresectable and/or metastatic angiosarcoma. Paclitaxel is the treatment of choice for advanced angiosarcoma [13]. Penel et al. reported that the progression-free survival (PFS) rate was 74% after two cycles of weekly paclitaxel treatment for unresectable angiosarcoma [14]. Fortunately, our two patients achieved good response to paclitaxel as first-line treatment. Some drugs, such as pazopanib and bevacizumab monotherapy, can be used as second-line treatment options. Recently, the efficacy of eribulin mesylate and trabectedin for angiosarcoma has been reported. Le Cesne et al. reported in a French retrospective study that the 3-month PFS rate when trabectedin was administered among angiosarcoma patients was 25% [15]. Further, treatment with eribulin mesylate helped achieve a 3-month PFS, 8.6-month OS, and 20% overall response rate in a prospective observational study [16].

## Conclusion

In conclusion, we experienced two cases of angiosarcoma that showed an early local recurrence 1 year after surgery, with or without positive surgical margins. Early detection of angiosarcoma and aggressive resection with adequately wide surgical margins will be required for achieving good local control and improving survival.

## Data Availability

The dataset supporting the conclusion of this article is included within the article.

## Abbreviations

AS: Angiosarcoma
BCS: Breast conserving surgery
RT: Radiation therapy
IDC: Invasive ductal carcinoma
